# Post-traumatic stress disorder in inpatient psychiatry staff: a narrative review

**DOI:** 10.3389/fpsyt.2025.1680477

**Published:** 2025-11-10

**Authors:** Caley Merritt, Vincent I. O. Agyapong, Nnamdi Nkire

**Affiliations:** Department of Psychiatry, University of Alberta, Edmonton, AB, Canada

**Keywords:** post-traumatic stress disorder, nurse, psychiatrist, inpatient, ward, psychiatry, nurses, physicians

## Abstract

**Introduction:**

Healthcare workers have high rates of post-traumatic stress disorder (PTSD). The aim of this study is to provide a narrative review of the literature on PTSD in psychiatrists, psychiatric nurses, and other healthcare staff working on psychiatric inpatient units.

**Methods:**

A search was conducted on MEDLINE for studies in English. Studies were included if they studied a population of inpatient psychiatric staff and had PTSD as an outcome measure. Of the 1487 articles the search returned, 26 were included.

**Results:**

Rates of post-traumatic stress ranged from 0% to 24%, depending on the measurement tool. Nurses had higher rates of PTSD compared to other psychiatric unit staff. There was no difference in PTSD prevalence between forensic and non-forensic psychiatric units. There were mixed findings on the association between gender and age and PTSD. Exposure to suicide, verbal aggression, physical violence, and other disturbing patient behaviors were associated with a higher risk of PTSD.

**Conclusion:**

High rates of post-traumatic stress are seen in healthcare workers in inpatient psychiatric settings. More research is needed on interventions to reduce PTSD in this population.

## Introduction

Post-traumatic stress disorder (PTSD) is a psychiatric condition that occurs when a person is exposed to a traumatic event and subsequently experiences intrusive re-experiencing of the event, avoidance of the event, negative alterations in cognition and mood, and alterations in arousal and reactivity (1). The preceding trauma can include directly experiencing or personally witnessing a traumatic event, learning that the traumatic event has happened to a close family member or friend, or experiencing repeated or extreme exposure to aversive details of traumatic events through one**’**s occupation (1). These symptoms must last for at least one month after the traumatic event in order to meet criteria for PTSD (1). If symptoms last less than one month, acute stress disorder is the more appropriate diagnosis (1). Symptoms can develop after a single traumatic event or as the result of chronic exposure to trauma. PTSD is associated with psychiatric comorbidity and increased mortality (1, 2). Not every traumatic event will lead to a diagnosis of PTSD, which is why it is important to look at risk factors that make people more vulnerable to developing PTSD after experiencing a traumatic event. A full diagnosis of PTSD may not be necessary to experience functional impairment. Studies have shown that people with symptoms of PTSD who do not meet the full diagnostic criteria still experience impairment from their symptoms and are at higher risk of suicide than those without PTSD symptoms (3, 4).

Certain occupations put workers at higher risk of witnessing or experiencing trauma. Healthcare is one of those occupations. Work-related trauma in healthcare workers has been studied extensively, with reported rates of PTSD ranging from 10% to 20% (5). In recent years, the focus of research on PTSD in healthcare workers has been related to the COVID-19 pandemic. Although the field of psychiatry has been relatively sheltered from the horrors of the COVID-19 pandemic, there are multiple facets of working with mentally ill patients that put mental health professionals at risk of PTSD. Although all healthcare workers are at higher risk of PTSD compared to the general population (6), psychiatric nurses, psychiatrists, and other staff working on inpatient psychiatric units are faced with a unique set of circumstances that could lead to traumatization. These can include physical and verbal aggression, patient suicide, or repeated exposure to other distressing situations.

### Violence and threats

Healthcare workers are more likely to experience violence and threats at work compared with most other professions (7). Violence can include physical aggression targeted at healthcare workers, patient-on-patient violence, or violent threats. Although violence can occur in any healthcare setting, psychiatric units and emergency departments have been found to have the highest risk of violence (8, 9). Staff in these settings may be asked to admit or care for violent or intoxicated patients. These patients may refuse to be interviewed or struggle when being asked to do things. They may direct their ire at staff verbally and in extreme cases lash out physically. In comparison with non-psychiatric nurses, mental health nurses experience more threats, assault attempts, vulgar behaviors, and physical assaults (10).

Being assaulted puts people at higher risk of PTSD (11). Psychiatric units contain unpredictable and mentally unstable patients, which may increase the risk of physical assault. Research has found that 84-88% of psychiatric nurses had been assaulted by a violent patient at some point in their career (12, 13). Injuries to nursing staff are not always secondary to targeted assaults. One study found that 62% of the reported injuries occurred while nursing staff was attempting to contain patient violence, while 38% were due to assault (14). Staff may be injured by a patient who is resisting going into a seclusion room, responding to command hallucinations or paranoid delusions, or experiencing akathisia or agitation as a side effect of medication. Trauma can be perpetuated when psychiatric unit staff have to provide ongoing care to the patients who assaulted them.

Verbal abuse and threats are frequent occurrences on inpatient psychiatric units and can be an additional source of trauma for mental health staff (15). A study conducted in a variety of mental health settings indicated that 61% of staff experienced emotional or psychological abuse (15). Both verbal abuse and threats are associated with higher likelihood of PTSD development (16).

### Patient death

Although encountering death is much less common in psychiatry than other areas of medicine, deaths do occur on psychiatric units. Deaths in the inpatient psychiatric setting can include deaths from medical causes, suicide, and homicide. Inpatient homicides are very rare, but can have severe mental health consequences for unit staff (17). Most psychiatric inpatient deaths are a result of medical causes, while a small proportion (5-7%) die by suicide (18, 19). Cardiovascular system disorders are the most common cause of death for psychiatric inpatients, with respiratory disorders as the second leading cause of death (18, 19). Although witnessing patient deaths from medical illnesses may be common in some areas of medicine, this is not a regular occurrence on psychiatric wards, thus these deaths may be experienced as more traumatic to psychiatric nurses.

Most mental health professionals encounter patient suicide during their years of practice (20–22). Prior research has found that 82% of psychiatrists, 47% of psychiatric trainees, and 55% of psychiatric nurses experience a patient suicide (20–22). In the inpatient psychiatric setting, exposure to suicide can mean directly witnessing the death by suicide, coming across the body during ward checks, or finding out that a patient died by suicide while on an approved outing from the unit or shortly after discharge. Although all patient suicides are distressing for psychiatric staff, certain types of patient suicides are associated with greater distress. The first suicide a person encounters may be associated with increased distress (21). Suicides encountered during training have a more severe impact than suicides encountered once a person becomes staff (23). Psychiatric trainees who felt responsible for the patient who died by suicide experienced more distress after the death (21). Suicide of a young person, suicide of a person with young children, and unexpected suicides, when the patient was future-oriented or seemingly improving, are more distressing for psychiatric trainees to encounter (21).

### Other workplace exposures

Inpatient psychiatry settings are high stress working environments. Psychiatric units are frequently loud and chaotic. Staff on psychiatric units may be exposed to repeated distressing patient behavior, such as self-harm or inappropriate sexual acts (24). Working on a psychiatric unit may also lead to vicarious trauma, which occurs when the emotional burden from repeatedly hearing the traumatic experiences of others leads to trauma-based symptoms that are similar to PTSD (24). Vicarious trauma used to be thought of as a distinct entity from PTSD, however changes from the DSM IV to the DSM V allow for diagnosing PTSD in the cases where people meet all of the PTSD symptom criteria (1). Inpatient psychiatry staff are also faced with challenging patient presentations. It can be distressing to work with patients with severe and persistent mental illness, even if the patients do not exhibit any aggressive behaviors.

Structural and organizational issues can pose challenges for inpatient psychiatric staff. Increased work stress is associated with increased risk of PTSD (25). Work stress can be increased due to interpersonal conflict with coworkers, limited autonomy, and excessive working hours (25). In a study of inpatient mental health nurses, the top work stressor identified was inadequate staffing (26). Inadequate staffing can lead to dangerous situations and nurses can experience personal distress from knowing that an individual patient**’**s care is being affected by lack of staff (26). Changes in the healthcare system were also noted to be a stressor, especially if staff were not notified in advance or consulted on the changes (26).

### Objectives

This paper aims to provide a narrative summary of the research on PTSD in nurses, psychiatrists, and other healthcare professionals who work on psychiatric inpatient units. The prevalence of PTSD, risk factors for development of PTSD, and interventions to reduce symptoms of PTSD in this population will be explored. Although there have been review articles published on PTSD in psychiatric nurses and other mental health staff (27), to our knowledge, there has never been a review article that focused on the inpatient psychiatric setting. We aim to synthesize the previous research on PTSD in psychiatric inpatient staff and identify gaps in the literature that could guide future research.

## Methods

### Study design

A narrative review was chosen to summarize the available literature on PTSD in psychiatric inpatient unit staff. Although narrative reviews do not provide the same rigorous and exhaustive search as systematic reviews, we chose this more flexible review format to provide an overview and thematic summary of the research on this topic. A narrative review was chosen to identify the relevant findings on PTSD in psychiatric inpatient staff and situate them within the broader context of PTSD research. The methodology for this review was developed through reading Ferrari’s (2015) and Green et al.’s (2006) articles on narrative reviews (28, 29). Since narrative reviews are more prone to selection bias, a structured search approach and strict inclusion and exclusion criteria were implemented to try to mitigate some of this risk.

### Search strategy

A literature search of the MEDLINE database through PubMed and Ovid using relevant terms and keywords to identify articles on the topic of PTSD in psychiatric inpatient staff. Publication date limitations were not set for the search. The search used MeSH terms “stress disorders, post-traumatic”, which included acute PTSD, chronic PTSD, delayed onset PTSD, moral injury, PTSD, post-traumatic neuroses, and stress disorder, and “health personnel”, which included healthcare professional(s), healthcare provider(s), healthcare worker(s), nurse(s), physician(s), and psychiatrist(s). The following keywords were used: “psychiatr*” OR (“mental health” AND “health personnel”) AND ((“stress” AND “disorders” AND “post traumatic”) OR “post-traumatic stress disorders” OR “ptsd” OR “psychological distress”). Titles were screened for eligibility according to the inclusion criteria. After screening by title, articles were exported to RefWorks and duplicate articles were removed. Articles were then screened by their abstracts. After exclusion of the articles that did not meet inclusion criteria based on their abstracts, the full texts of the remaining articles were assessed for eligibility. A hand search of the references of the included articles was then conducted to identify any additional relevant studies.

### Inclusion and exclusion criteria

Inclusion criteria for this review were studies/papers with a focus on (a) inpatient psychiatric unit setting, (b) study population of mental health nurses, psychiatrists, psychiatry residents, or other healthcare workers on psychiatric units, (c) outcome measure of PTSD or PTSD symptoms measured by a valid measurement tool, and (d) primary research. Exclusion criteria were (a) full text not available online, (b) language other than English, (c) setting not specified as psychiatric inpatient ward or psychiatric hospital, (d) secondary research, case studies, or exclusively qualitative studies, and (e) studies that did not include PTSD or PTSD symptoms as an outcome measure. Studies were excluded if they used a population of healthcare workers from both inpatient and outpatient settings and did not separate them in the results.

### Data extraction

Data was extracted by one author (C.M.). For each study, the following data was extracted and added to a table: first author, year of publication, country of study, sample population, sample size, study design, PTSD measurement tool, PTSD prevalence, and predictors of PTSD.

## Results

### Search outcomes

**Figure 1:**
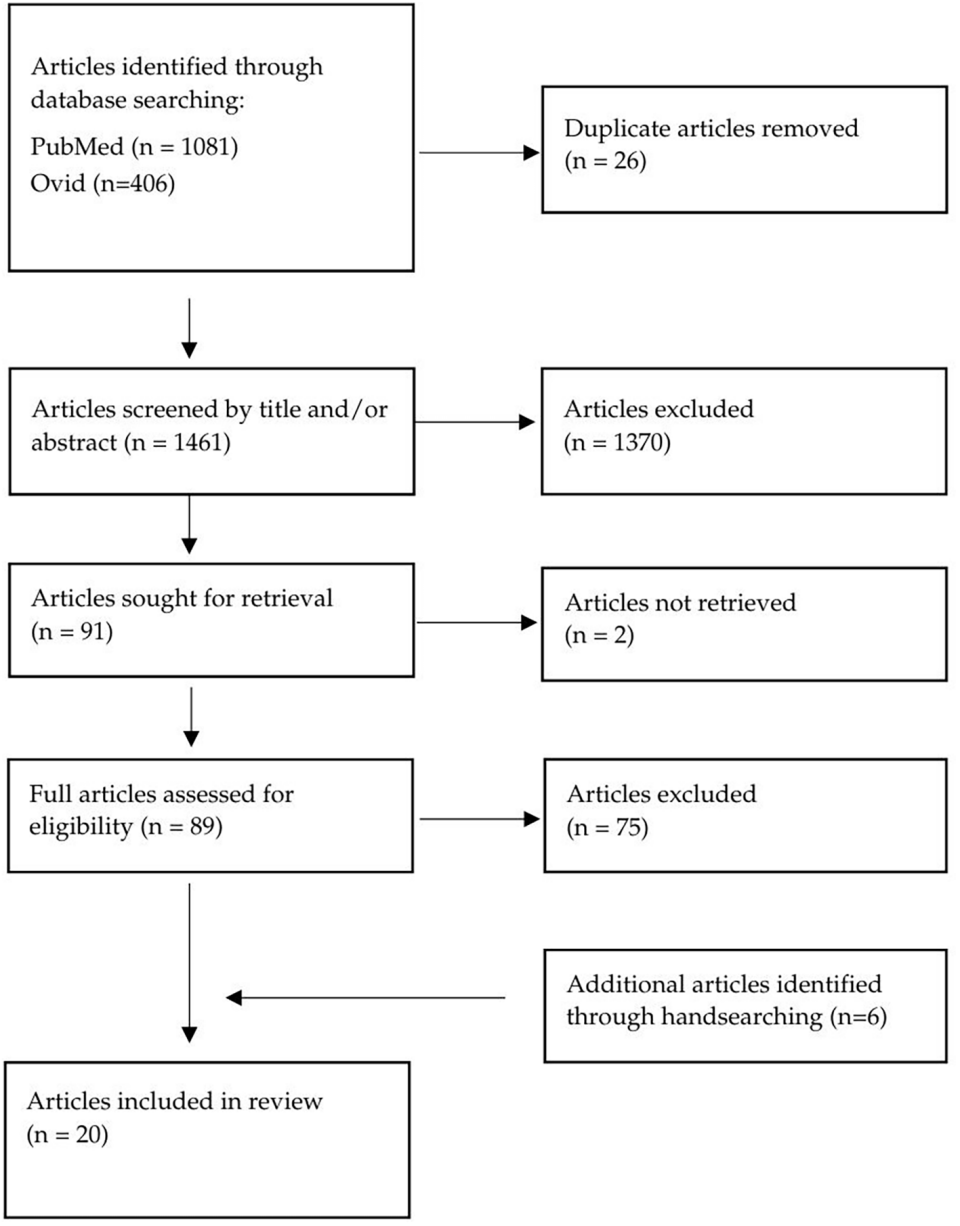
Flow diagram of article selection process.

The process of study selection is outlined in the flow-chart (**Figure 1**). The electronic database search was conducted by one author (C.M.) in July 2024. The database search identified 1487 articles. The citations were exported to RefWorks and 26 duplicate articles were removed. The abstracts of 1461 articles were reviewed, and 1370 articles were excluded for not meeting the inclusion criteria. Two articles were unable to be retrieved. The remaining 89 articles were assessed for eligibility by reviewing the full texts. Fourteen articles met the inclusion and exclusion criteria after full-text review. The main reasons for study exclusion were not having PTSD as an outcome measure (n=46) and the population including more than just inpatient psychiatric staff (n=26). Six additional records were identified through handsearching the references of the eligible articles and included in the review.

### Study characteristics

The characteristics of the 20 articles included in this review are presented in **Table 1**. Included studies were published between 2003 and 2022. Studies were conducted in a variety of locations including Canada, USA, UK, Denmark, Norway, Switzerland, Germany, Botswana, Iran, Israel, Japan, China, and Australia. There was significant variation in sample size, with sample sizes ranging from 35 to 2678. Eleven studies included multiple types of healthcare workers, including nurses, patient care assistants, psychiatrists, social workers, psychologists, clinical managers, security, housekeeping, and nutrition services. Nine studies included only mental health nurses. Most of the studies were cross-sectional (n=16, 80%), three were prospective cohort studies (15%), and one was a randomized control trial (5%).

**Table 1:** Summary of studies assessing PTSD in healthcare professionals on psychiatric units.

Reference	Country	Inpatient setting	Sample	Sample size	Study design	PTSD measure	PTSD prevalence
Al Ali et al. (2020) (42)	Denmark	18 psychiatric units	Staff who had experienced workplace violence	250	Cohort	HTQ (Danish version)	8%
Andersen et al. (2018) (38)	Denmark	Psychiatric wards (one of four areas of human service work examined in this study)	Psychiatric staff	548 psychiatric staff	Cohort	IES-R	2011 – 14% 2015 – 17%
Chen et al. (2008) (43)	Taiwan	A psychiatric hospital	Nurses, nursing aids, and clerks	222	Cross-sectional	ILO/ICN/WHO/PSI Questionnaire	10%
Cramer et al. (2020) (57)	United Kingdom	Three secure mental health correctional facilities	Nurses, nursing assistants, psychologists, and psychiatrists	170	Cross-sectional	PCL-C	Not reported
Hilton et al. (2017) (46)	Canada	Forensic and non-forensic units at a psychiatric hospital	Nurses, allied health professionals, housekeepers, and food service workers	219	Cross-sectional	PCL-C	24%
Hilton et al. (2020) (53)	Canada	Forensic and non-forensic units at three psychiatric hospitals	Nurses, physicians, allied health professionals, clinical managers, and staff who worked with inpatients in off-ward areas	761	Cross-sectional	PCL-5	16%
Inoue et al. (2006) (39)	Japan	Two psychiatric hospitals	Nurses	225	Cross-sectional	IES-R	21%
Jacobowitz et al. (2015) (55)	United States	A psychiatric hospital	Nurses, psychiatric aides, assistant counselors, psychiatrists, case coordinators, and therapeutic rehabilitation specialists	172	Cross-sectional	PCL-C	Not reported
Lauvrud et al. (2009) (51)	Norway	A high security forensic psychiatric unit	Nurses	70	Cross-sectional	PCL-C	0%
Lee et al. (2014) (47)	Australia	Psychiatric units at four general hospitals and forensic units at a secure forensic psychiatric hospital	Nurses	196	Cross-sectional	PCL-C	Severity scoring method: Forensic – 17% Non-forensic – 18% Symptom endorsement scoring method: Forensic – 12% Non-forensic – 16%
Lee et al. (2015) (48)	Australia	A secure forensic psychiatric hospital	Nurses	Timepoint 1: 97 Timepoint 2: 107	Cross-sectional	PCL-C	Severity scoring method: Timepoint 1 – 17% Timepoint 2 – 18% Symptom endorsement scoring method: Timepoint 1 – 12% Timepoint 2 – 11%
Liu et al. (2022) (49)	China	Three psychiatric hospitals	Nurses	351	Cross-sectional	PCL-C	18%
Newman et al. (2019) (40)	Australia	A forensic hospital	Nurses, physicians, allied health, and administrative staff	218	Cross-sectional	IES-R	Symptoms of clinical concern for PTSD – 25% Probable PTSD – 10%
Nhiwatiwa (2003) (58)	United Kingdom	Four psychiatric hospitals	Nurses who had been assaulted by patients	40	Randomized control trial	IES	Not reported
Olashore et al. (2018) (52)	Botswana	A psychiatric hospital	Nurses, student nurses, psychiatrists, medical officers, psychologists, and allied health staff	201	Cross-sectional	PCL-C	18%
Richter & Berger (2006) (41)	Germany	Nine state psychiatric institutions	Nurses, physicians, social workers, and housekeeping staff who had been assaulted by a patient	35	Cohort	PCL-C	2 months post-assault – 9% 6 months post-assault – 11%
Seto et al. (2020) (54)	Canada	Forensic and non-forensic inpatient psychiatric units at three psychiatric hospitals	Nurses, patient care assistants, psychiatrists, social workers, psychologists, clinical managers, security, housekeeping, and nutrition services	761	Cross-sectional	PCL-5	16%
Takahashi et al. (2011) (22)	Japan	Six psychiatric hospitals and two general hospitals	Psychiatric nurses	531	Cross-sectional	IES-R	14%
Tirgari et al. (2019) (56)	Iran	Two hospitals and one rehabilitation center	Psychiatric nurses	160	Cross-sectional	PCL-C	Not reported
Zerarch & Shalev (2015) (44)	Isreal	Two psychiatric hospitals (in comparison with community health clinics)	Psychiatric nurses	90 psychiatric staff	Cross-sectional	PTSD Inventory	7%

HTQ, Harvard Trauma Questionnaire; IES, Impact of Event Scale; IES-R, Impact of Event Scale-Revised; ILO/ICN/WHO/PSI Questionnaire, International Labour Organization, International Council of Nurses, World Health Organization, and Public Services International’s Health Sector Workplace Violence Questionnaire; PCL-C, PTSD Checklist – Civilian; PCL-5, PTSD Checklist-5.

A variety of PTSD measurement tools were used in the included studies, as presented in **Table 1**. Half of the included studies used the PTSD Checklist – Civilian (PCL-C) (n=10, 50%), while two used the newer PTSD Checklist-5 (PCL-5) (10%). One study used the Impact of Event Scale (IES) (5%), while four studies used the newer Impact of Events Scale – Revised (IES-R) (20%). Each of the following measurement instruments were used by one study (5%): PTSD Inventory, Harvard Trauma Questionnaire (HTQ), and the ILO/ICN/WHO/PSI Questionnaire. The different measurement instruments for PTSD were created based on different diagnostic criteria for PTSD. The Impact of Events Scale developed prior to the inclusion of PTSD in the Diagnostic and Statistical Manual for Mental Disorders (DSM) (30), which occurred with the introduction of the DSM III in 1980. The remaining measurement tools were based on the PTSD criteria in different iterations of the DSM. The HTQ is based on the DSM-III-R (31). The PTSD Inventory, the ILO/ICN/WHO/PSI Questionnaire, the Impact of Events Scale – Revised, and the PCL-C are all based on the DSM-IV (32–35). The PCL-5 is based on the DSM-V diagnostic criteria for PTSD (36).

### PTSD prevalence

Prevalence of PTSD in psychiatric inpatient unit staff was reported in 16 of the included studies, as shown in **Table 1**. Rates of PTSD varied based on the measurement tool used. For the IES-R, scores ≥ 24 indicate symptoms that are clinically concerning for PTSD (37) and scores ≥ 33 indicate a probable diagnosis of PTSD (32). In the studies using the IES-R, symptoms of clinical concern for PTSD were found in 14-25% of the study sample (22, 38–40) and probable PTSD was found in 10% of the one study that used the ≥ 33 cut-off (41). The studies that used the PTSD Inventory, HTQ, and ILO/ICN/WHO/PSI Questionnaire found that 7-10% of their sample met criteria for PTSD (42–44). The PCL-C has two different scoring methods. In the severity scoring method, total scores ≥ 44 indicate a diagnosis of PTSD (45). Studies that used the severity scoring method found that 17-24% of their samples met criteria for PTSD (46–49). In the symptom endorsement scoring method, PTSD is diagnosed if an individual endorses one re-experiencing symptom, three avoiding and numbing symptoms, and two hyperarousal symptoms (50). Endorsement indicates a score of 3 or more on that item (50). The studies that used the symptom endorsement scoring method found that 0-18% of their samples met criteria for PTSD (41, 47, 48, 51, 52). The two studies using the PCL-5 used a cut-off of 33 to indicate probable PTSD, which was identified in 16% of participants in both studies (53, 54). One of the studies that used the PCL-5 also used a more stringent criteria to indicate a diagnosis of PTSD (53). The more stringent criteria for the PCL-5 includes reporting of any critical event, score ≥ 33, at least one symptom in each symptom cluster, symptom duration of over one month, and functional impairment (53). The study using the more stringent scoring method for the PCL-5 found that 9% met criteria for PTSD (53).

### Risk and resilience factors associated with PTSD

**Table 2** displays findings from the included studies on risk factors and protective factors for PTSD.

**Table 2:** Factors associated with PTSD development in psychiatric inpatient staff.

Reference	Factors associated with PTSD development in psychiatric inpatient staff
Al Ali et al. (2020) (42)	• Diagnosis of acute stress disorder after an incident of workplace violence • Female gender • Lower social support
Andersen et al. (2018) (38)	• Work-related threats occurring within the last 12 months • Work-related threats occurring 4–5 years ago • Work-related violence occurring within the last 12 months
Cramer et al. (2020) (57)	• Preference to avoid emotional stimuli and suppress emotional content
Hilton et al. (2017) (46)	• Job position of nurse • Exposure to a higher number of disturbing patient behaviors • Exposure to patients who are constantly screaming • Exposure to patients who are hording • Exposure to patients who are physically resisting care • Exposure to patients inflicting self-injury • Exposure to patients eating harmful non-food items • Exposure to patients drinking from the toilet • Exposure to patients who flood their room • Experiencing patient elopement
Hilton et al. (2020) (53)	• Job position of nurse • Exposure to a higher number of critical events at work • Exposure to any individual critical event (physical assault by a patient (with or without injury to victim), injury while restraining patient, sexual assault by patient, threat of death or serious injury to staff or staff’s family, violent or accidental health, and suicide or near fatal attempt) • Exposure to a higher number of chronic stressors at work • Exposure to any individual chronic patient stressor (damaging the room, drinking from the toilet, eating harmful items, elopement, flooding the room, hoarding, physically resisting care, screaming constantly, self-injury, public sexual behavior, smearing feces, wandering, verbal abuse or threats, and physical violence)
Inoue et al. (2006) (39)	• Dissatisfaction with familial support • Neuroticism • Younger age
Jacobowitz et al. (2015) (55)	• Burnout • Younger age • Experiencing a greater number of severe physical attacks
Lauvrud et al. (2009) (51)	• Longer duration of nursing experience • Low compassion satisfaction
Lee et al. (2015) (48)	• Working on a unit where a murder occurred
Liu et al. (2022) (49)	• Job stress
Newman et al. (2019) (40)	• Vicarious trauma
Olashore et al. (2018) (52)	• Exposure to violence in the last 12 months • Neuroticism
Richter & Berger (2006) (41)	• Experiencing a severe injury from a patient assault
Seto et al. (2020) (54)	• Exposure to trauma at work • Male gender • Comorbid depression • Comorbid anxiety
Tirgari et al. (2019) (56)	• Higher compassion fatigue • Lower compassion satisfaction • Higher burnout

#### Employment factors

Work setting can have an impact on the development of PTSD. Psychiatric inpatient staff were found to have a higher PTSD prevalence than community nurses and staff in care homes for the elderly (38, 44). Interestingly, two studies found no difference in PTSD prevalence between forensic and non-forensic mental health staff, (46, 47), while one study found a lower prevalence of PTSD in psychiatric inpatient staff compared to employees working in prison and probation services (38).

Job position can also impact PTSD prevalence. Nurses working on psychiatric units were found to have higher rates of PTSD than physicians, allied health professionals, clinical managers, housekeepers, and food service workers (46, 53).

Findings in the current review were mixed regarding the association between duration of employment and PTSD. Two studies found that length of work in the psychiatric department was not significantly associated with PTSD symptoms (39, 44), while one study found that those with more years of nursing were more likely to meet criteria for a diagnosis of PTSD (51). Higher job stress, burnout, and vicarious trauma were associated with greater number and severity of PTSD symptoms (40, 49, 51, 55, 56).

Exposure to distressing events is a common experience for those working on psychiatric units. Most inpatient psychiatric staff have been exposed to violence or threats of violence (46, 51–53). Experiencing work-related violence or threats, especially within the past 12 months, puts psychiatric inpatient staff at higher risk of PTSD (38, 48, 52–55). One study found that assaulted staff with severe injuries were most likely to meet criteria for a diagnosis of PTSD (41). Second most likely were staff that had no injuries (41). Least likely were those with mild to moderate physical injuries. (41). One would have thought that patients with no injuries would be less likely to experience PTSD than those with mild to moderate physical injuries. This line of thinking, however, negates the impact of the severity of the fear factor and psychological scars individuals experienced at the time of exposure to the trauma, which do not necessarily correlate to the degree of physical injuries. It is important to monitor people after a violent incident, regardless of injury, because diagnosis of acute stress disorder is a predictor for PTSD development (42).

Even non-violent behavior can be distressing for staff to witness repeatedly. Examples of such behaviors are feces smearing, self-injury, inappropriate sexual behavior, constant screaming, physically resisting care, and having to restrain uncooperative patients (46). Almost all psychiatric staff had been exposed to these disturbing behaviors (46). Higher number and severity of PTSD symptoms were associated with a greater number of disturbing behaviors experienced (46, 53).

#### Personal factors

There were mixed results on the association between gender and PTSD, with one study reporting that female healthcare workers on psychiatric units are more likely to meet criteria for a diagnosis of PTSD (42), one study reporting that male healthcare workers on psychiatric units are more likely to screen positive for PTSD (54), and three studies finding no correlation between gender and PTSD (39, 44, 53). One study found that older mental healthcare workers reported more PTSD symptoms (56), while two studies found that younger staff were more likely to screen positive for PTSD (39, 55). Marital status was not found to be associated with PTSD (39, 44).

It is well known that there are high rates of comorbidity between psychiatric conditions (1). One study included in this review found that approximately half the psychiatric inpatient staff who met the screening cutoff for PTSD also met the cutoff for anxiety or depression (54). Past trauma history may also be relevant. One study found that 60% of mental health hospital staff had a personal trauma history (40). Psychiatric staff with a history of trauma were found to have a higher number of PTSD symptoms (40).

Social support is also related to PTSD. In two studies, healthcare workers who had less social support and lower perceived familial support were more likely to screen positive for PTSD (39, 42).

Personality factors were also associated with PTSD symptoms. Higher neuroticism is associated with more symptoms of clinical concern for PTSD in psychiatric hospital staff (39, 52). People who prefer to experience and express their emotions are less likely to experience PTSD symptoms than those who avoid experiencing and expressing their emotions (57).

### Interventions to reduce PTSD among psychiatric inpatient staff

Only one interventional study met inclusion and exclusion criteria for this review, as shown in **Table 3**. Nhiwatiwa et al. (2003) found that the brief educational intervention of reading a booklet on the effect of trauma and coping in the direct aftermath of a patient assault was associated with greater PTSD symptoms in nurses who had been assaulted (58).

**Table 3:** Interventional studies on PTSD in healthcare professionals on psychiatric units.

Reference	Setting	Participants	Sample size	Study design	Intervention	PTSD measure	Findings
Nhiwatiwa (2003) (58)	Four Psychiatric Hospitals in the United Kingdom	Nurses who had been assaulted by patients	40	RCT	Reading a booklet on the effect of trauma and coping mechanisms	IES	Those who received the brief educational intervention had more PTSD symptoms at the three-month follow-up than those who did not receive the intervention.

IES, Impact of Event Scale; RCT, Randomized Control Trial.

## Discussion

### PTSD prevalence

A wide range of PTSD rates were found in the 16 studies included in this review that measured PTSD prevalence. The lifetime prevalence of PTSD is around 4% in the general population and 6% in people who have been exposed to trauma (59). All but one of the studies included in this review found a higher prevalence of PTSD in psychiatric inpatient staff (7-25%) than the general population. This review found a lower PTSD prevalence in staff working on psychiatric units (7-25%) compared to three recent meta-analyses of PTSD prevalence of healthcare workers and nurses during the COVID-19 pandemic, which found overall pooled prevalences of 22%, 29%, and 34% (60–62). Rates of PTSD development after a traumatic event differ depending on the specific trauma experienced, with sexual violence and homicide being the most likely to lead to a diagnosis of PTSD (63). It is not surprising that studies on nurses in the COVID-19 pandemic found higher rates of PTSD than the present study, given the nurses would have the general stressors associated with their job position plus the added stressor of the pandemic.

One study in this review, Lauvrud et al. (2009), found that none of the psychiatric nurses in their sample met criteria for PTSD (51). The discrepancy between this finding and the other studies included in this review cannot be accounted for by differences in measurement tool. Lauvrud et al. (2009) used the symptom endorsement scoring method for the PCL-C, which was used by four other studies including in this review that found PTSD prevalence from 9% to 16% (41, 47, 48, 52). Lauvrud et al. (2009) hypothesized that the low prevalence of PTSD symptoms was due to unique characteristics of their clinic, which was a high security forensic psychiatry unit in Norway that has a strong collegial spirit and a very high staff to patient ratio (5:1) (51). Notwithstanding the results from Lauvrud et al. (2009), the high rate of PTSD in psychiatric inpatient staff identified in this review suggests the need to target this population with efforts to mitigate the risk of PTSD development.

### Demographic factors

Female gender is a well-known risk factor for PTSD in the general population (64). In an umbrella review of PTSD risk factors that included 33 systematic reviews and meta-analyses, female gender was found to be one of the most robust risk factors in the development of PTSD (64). Surprisingly, the results related to gender and PTSD symptoms in psychiatric inpatient staff were mixed. Three studies found no association between gender and PTSD, (39, 44, 53), one study found that male psychiatric staff were more likely to screen positive for PTSD compared to female staff (54), and one study found female psychiatric staff had a higher prevalence of PTSD than male staff (42). Seto et al. (2020) suggested some reasons for the discrepant finding that male staff were more likely than female staff to screen positive for PTSD. First, they suggested that male healthcare workers on psychiatric units may be exposed to more violence than female healthcare workers due to being selected to manage the most aggressive patients (54). Second, Seto et al. (2020) suggested the increased PTSD symptoms in male staff could relate to social support, as they found that male staff utilized less formal mental health supports and had more attitudinal barriers to accessing support than female staff (54). Interestingly, a recent systematic review and meta-analysis of sex and gender differences in risk factors for PTSD found that the higher PTSD symptom severity in women is due to the higher psychological stress response immediately after a traumatic event, particularly symptoms of acute stress disorder, anxiety, and depression (65). One might wonder if there is something about female psychiatric staff that mitigates this risk. Perhaps, a greater knowledge of the psychological reactions that occur post-traumatic experience due to their work experience. This would be consistent with prior research that has found that the gender difference in PTSD may be less pronounced when specific professional training has been completed (66).

The findings related to age and PTSD in this review were also mixed, with one study finding that older psychiatric nurses report more PTSD symptoms (56) and two studies finding that younger psychiatric staff report more PTSD symptoms (39, 55). In the general population, younger age at the time of a traumatic event is a risk factor for the development of PTSD (67). However, a systematic review of healthcare workers found that older age predicts PTSD symptoms (66). This finding was thought to be a result of the cumulative effect of workplace violence over time, which was consistent with their finding that healthcare workers with longer length of service have higher rates of PTSD (66). The findings of the current review do not necessarily support this because only one study found that longer duration of nursing experience was associated with greater likelihood of PTSD diagnosis (51), while two studies found no association (39, 44). It is possible, however, that the increased risk from younger age is mitigated by the lower length of nursing experience and the increased risk from longer duration of nursing experience is mitigated by older age, thus leading to non-significant results in correlational studies.

### Psychological factors

History of a diagnosed psychiatric condition is a well-known risk factor for future mental health concerns (66, 67). History of depression, anxiety, PTSD, emotional dysregulation, and sleep difficulties predispose people to the development of PTSD (66, 67). Only one study included in this review addressed psychiatric diagnoses other than PTSD and this was approached from the comorbidity perspective (54). Seto et al. (2020) found that people were more likely to screen positive for PTSD when they also screened positive for anxiety or depression (54). None of the included studies investigated history of diagnosed mental health condition as a risk factor for PTSD development.

Only one of the included studies addressed trauma history. This study found that previously experiencing a traumatic event increases risk of PTSD after experiencing an additional traumatic event (40). This is consistent with a 2023 meta-analysis that found that history of trauma is a risk factor for PTSD development following subsequent trauma exposure exposure (67). Only one study included in this review attempted to control for trauma history. Jacobowitz et al. (2015) measured past traumatic experiences with the Life Events Checklist and using an adjusted PCL-C score for their PTSD measure (55). This study did not report PTSD prevalence using either the raw PCL-C score or the adjusted PCL-C score. Three of the included studies instructed participants to fill out the PTSD measure thinking about a traumatic exposure in their current workplace (40, 46, 54). It is unclear how well people are able to separate out PTSD symptoms if they experience both a personal and a work-related traumatic event. Interestingly, Olashore et al. (2018) excluded participants if they had a history of PTSD (52).

Personality factors can also contribute to likelihood of PTSD development after experiencing a traumatic event. Two studies in this review found that higher neuroticism was associated with screening positive for PTSD (39, 52). Although the correlational data in the two studies included in this review cannot identify a causal relationship, these findings are supported by a recent meta-analysis that found that pre-trauma neuroticism was a strong predictor of PTSD symptoms (67).

### Job position

Both the studies included in this review that compared different types of psychiatric staff found that nurses have higher rates of PTSD than physicians, allied health professionals, clinical managers, housekeepers, and food service workers (46, 53). This is consistent with the findings of a recent meta-analyses on PTSD in healthcare workers during the covid-19 pandemic that also found higher PTSD prevalence in nurses compared to other healthcare staff (68). The higher rate of PTSD in nurses is thought to be due to higher exposure to critical events and disturbing behavior by patients due to a higher proportion of direct clinical care compared to allied health and other staff (46, 53).

### Forensic vs. non-forensic setting

Both forensic and non-forensic inpatient psychiatric settings were included in the current review. Two studies included in this review compared PTSD rates on forensic and non-forensic units (46, 47). Hilton et al. (2017) found no difference in PTSD prevalence between staff working on forensic units and non-forensic units at the same psychiatric hospital in Canada (46). They also found no difference in rates of exposure to violence or other disturbing behaviors between the two units (46). Lee et al. (2014) found no difference in PTSD prevalence between nurses working on inpatient psychiatric units in general hospitals and nurses working on inpatient units in a forensic mental health hospital in Australia (47). They also found no difference in exposure to actual, attempted, or threatened violence between the forensic and non-forensic mental health nurses (47). The similar rates of violence between forensic and non-forensic settings in both the included studies in this review is inconsistent with past research, which shows higher rates of violence towards nurses in forensic compared to general medical settings (69). This discrepancy may be is due to how violence is conceptualized and measured in the studies. The two studies that compared forensic and non-forensic psychiatric inpatient settings in this review included verbal aggression in addition to physical aggression in their definition of violence (46, 47). Another possible explanation for this finding is that there are more similarities between forensic settings and inpatient psychiatry settings than other areas of medicine. For example, both forensic and non-forensic psychiatry wards have on-unit security guards, secure locked rooms, and most patients are being held involuntarily. One study in this review compared inpatient psychiatric staff to staff working in prison and probation services and found that those working in prison and probation services have higher rates of PTSD (38). This finding is not necessarily comparable to the other studies on forensic settings included in this review, because those working in prison and probation services may not be healthcare workers and may be working with the general prison population rather than the forensic psychiatry population.

### Future research directions

One of the aims of this review was to identify any gaps in the current literature on PTSD in psychiatric inpatient staff. Multiple gaps in the literature were identified.

None of the including articles in this review assessed for prior mental health conditions. Depression, anxiety, and other psychiatric conditions are known risk factors for PTSD (11). Future research should assess psychiatric history and, more specifically, a history of PTSD in the sample of psychiatric staff being studied. It would be important to investigate how previous diagnosis of PTSD impacts severity of post-traumatic symptoms and likelihood of meeting criteria for PTSD again after experiencing a traumatic event while working on the inpatient psychiatric unit. Identifying the preexisting prevalence of PTSD in people who choose to work in the mental health field could be beneficial to put the PTSD rates reported in this review into context. It is possible that people with a history of trauma may be more inclined to work in the field of psychiatry.

Future research could explore the relationship between patient suicide and PTSD in psychiatric unit healthcare workers. Although there are many articles on how patient suicide affects healthcare workers, we were only able to find one that was specific to psychiatric inpatient unit staff (22). Given the relatively high frequency of suicide in the inpatient psychiatric setting, this is an important area to focus future research on.

Only one study included in this review was interventional. This highlights a clear need for research into any interventions that could be helpful in this population. A 2021 systematic review and meta-analysis found that cognitive behavioral therapy (CBT) and mindfulness-based stretching and deep breathing exercise were effective psychological interventions for reducing PTSD symptom severity in healthcare providers with PTSD (70). Randomized control trials of cognitive-behavioral or mindfulness-based interventions would be highly clinically relevant in a population of psychiatric inpatient staff. In non-healthcare worker populations, meta-analyses have found that effective psychological treatments for PTSD include cognitive therapy, exposure therapy, and eye movement desensitization and reprocessing (EMDR), and effective pharmacological treatments for PTSD include certain antidepressants, antipsychotics, and anticonvulsants (71–75). There is no single preferred treatment in the treatment of PTSD, so a variety of treatments should be studied in psychiatric inpatient healthcare workers to address the significant gap in the current literature.

### Implications for policy and practice

Based on the factors associated with PTSD identified in this review, we have outlined some interventions that may be useful to study in this population based on previous research in similar populations.

#### Pre-trauma interventions

Given the high correlation between being assaulted at work and experiencing PTSD symptoms, reducing patient violence on inpatient psychiatric units could lead to lower rates of PTSD. Measures that reduce the risk of violence towards healthcare staff include presence of a security guard, applying penalties to violent patients to deter others from violence, adequate staffing, and programs to improve staff communication (76, 77).

Many healthcare workers experience the workplace culture that violence is an expected part of the job (78). This culture can lead to people feeling unsafe in their work environment. Higher perceived safety of the workplace is associated with lower rates of PTSD symptoms (5). When violence is accepted as part of the job, violent incidents may be more likely to go unreported (79). Staff must not only have the means to report violent incidents, but must also believe that reporting the incident will lead to changes being implemented by management (77). Management can instill this confidence in their staff by following up on the incidents and providing support to the staff who experienced the violence (80).

Multiple studies included in this review found that job stress is associated with higher rates of PTSD (40, 49, 51, 55, 56). Efforts should be made to implement policies and programs that reduce work stress for mental health employees (49). Stress reduction in the workplace can include improved organizational support, increased internal cohesion, and reduced workload (49). Given that burnout is associated with greater PTSD symptoms (55, 56), reducing staff burnout may also serve to combat PTSD development. A 2023 review article on interventions to reduce burnout for nurses, physicians, and allied healthcare professionals found that both individually focused interventions and organizationally focused interventions can reduce burnout (81). For the individually focused interventions, mindfulness-based interventions were consistently shown to reduce burnout (81). Organizationally focused interventions that can reduce burnout include reduced workloads and a peer support network (81).

#### Post-trauma interventions

In the past, critical incident stress debriefing was used in the aftermath of traumatic events. Critical incident stress debriefing was developed in the 1980s with the aim of reducing the likelihood of PTSD development after a traumatic event through validating and normalizing peoples’ response to trauma. Research has consistently shown that this is not an effective method for preventing or reducing the likelihood of PTSD (82). The one interventional study included in this review found that the brief educational intervention of reading a booklet on the effect of trauma and coping in the direct aftermath of a patient assault was associated with greater PTSD symptoms in nurses who had been assaulted (58), which is a finding consistent with past research (82).

It is clear from prior research that critical incident debriefing is at best ineffective and at worst harmful, but there are other interventions that can be implemented after a traumatic event to reduce the risk or severity of PTSD symptoms in staff. A review article on interventions for healthcare workers with PTSD found that both cognitive behavioral therapy and mindfulness-based interventions reduced PTSD symptoms (83). Gerhart et al. (2016) found that an eight week program on mindfulness and communication training led to a reduction in the re-experiencing symptoms of PTSD in palliative care providers (84). Mindfulness-based exercise programs have also been found to reduce PTSD symptom severity in healthcare workers (85). Other interventions shown to reduce PTSD symptoms in healthcare staff are emotional regulation training and resilience-building programs (86, 87).

Both formal and informal supports are important in the prevention of PTSD. Formal supports include workplace policies and programs, support groups, and appointments with healthcare professionals such as therapists, psychiatrists, and family physicians. Occupational support is often felt to be insufficient after a traumatic event. Studies have found that a minority of nurses receive psychological supports after a patient suicide, but most nurses thought it would be helpful (22, 88). Psychiatric nurses who experienced an inpatient suicide had decreased anxiety and depression when a formal peer support group was implemented (88). One study in this review addressed how often psychiatric unit staff were utilizing formal supports (54). The highest utilized formal support was family physicians, with 47% of psychiatry unit staff indicating that they had seen their family physician in the last year (54). The lowest utilized formal support was addiction services, with 4% of psychiatric unit staff seeking these supports (54). Intermediately utilized formal supports included online material (36%), printed material (33%), medication (30%), therapy (25%), and employment services (20%) (54). The overall utilization of formal mental health supports are low and people may benefit from encouragement from their employers to utilize these services.

In addition to increasing the formal supports offered, it may also be useful to decrease the barriers that people face to seeking support. In a study of psychiatric unit staff, 56% reported attitudinal barriers to seeking support and 38% reported structural barriers (54). The most commonly reported attitudinal barriers were thinking that they could handle the problem without treatment, not wanting others to find out that they were getting treatment, and worrying that mental health treatment could have a negative impact on their job (54). Employees should be reassured that accessing mental health services will be confidential and will not have negative outcomes for their career. The main structural barriers to seeking formal support were not having enough time and not having coverage for services (54). Workplaces could ensure that mental health supports were covered, especially in the wake of a traumatic patient experience such as an assault or suicide.

Two studies included in this review found that low informal social support was associated with more symptoms of PTSD (39, 42). Informal support generally involves unstructured emotional support from family, friends, and colleagues. Past research has shown that informal supports are more helpful for patients in the aftermath of workplace trauma and are utilized at a much higher rate than formal supports (89). Research has found that for resident doctors, support from their co-residents is more important than support from their supervisors (90). For junior doctors, many of whom have to relocate for residency, higher rates of mental disorders were found in residents who were working in a location where they did not have family or friends (90). Researchers suggested improving informal social supports for junior doctors by hosting informal social gatherings and limiting work hours to allow residents time to develop friendships outside of the work setting (90).

### Limitations

There are significant limitations pertaining to the methodology of this review. This review would have been more scientifically rigorous if a second author independently conducted study selection and data extraction. Having only one author complete study selection and data extraction increases the risk of bias in study selection, misapplication of inclusion and exclusion criteria, and errors in data extraction. Only one database (MEDLINE) was used for the search which increases the risk of missing relevant research on the review topic. Only including studies published in English and studies with the full-text available online further increases the risk of missing relevant studies.

Another limitation is the way that post-traumatic stress is measured. Cut-off points for PTSD often vary between different studies, making it challenging to compare rates of PTSD symptomatology. The studies included in this review measure PTSD symptoms based on self-report questionnaires. Self-report measures are more prone to bias due to their subjective nature. Although these are frequently used in research, they can only comment on PTSD symptoms and not a clinical diagnosis of PTSD. PTSD must be diagnosed with a full diagnostic interview, which is challenging to implement in the research setting. Surprisingly, a recent umbrella review of PTSD prevalence did not find a statistically significant difference between PTSD prevalence in studies that used structured interviews compared to self-report measures (63). However, nine of the 16 meta-analyses included in the umbrella review found that structured clinical interviews led to lower PTSD prevalence than self-report questionnaires (63). Additionally, measurement tools often lag behind diagnostic criteria. The majority of studies included in this review used measurement tools that were based on the DSM-IV diagnostic criteria. Only two studies used the PCL-5 which is based on the DSM-V diagnostic criteria for PTSD. There were substantial changes to the PTSD diagnostic criteria between the DSM-IV and the DSM-V including the addition of a recurring exposure as a possible traumatic event and the addition of a fourth diagnostic cluster of negative alterations in mood and cognition. As further research is published, it will be easier to compare prevalence rates across studies using the same diagnostic criteria.

## Conclusions

Healthcare workers on inpatient psychiatric units are at high risk of development of PTSD. The findings of this review underscore the urgent need for systemic policy and practice changes to protect the mental health of staff working in psychiatric inpatient settings. High rates of assault and exposure to disturbing behavior—regardless of whether staff work in forensic or non-forensic units—highlight the importance of violence prevention strategies. Policies must move beyond accepting violence as “part of the job” and instead prioritize creating a culture of safety through measures such as 24/7 security presence, adequate staffing, staff training in de-escalation, and consistent reporting procedures. Reporting must be normalized, with clear pathways for follow-up and accountability, to ensure staff feel supported and empowered. Furthermore, training healthcare professionals in coping strategies before traumatic events occur—such as distress tolerance and trauma-informed care—can serve as a protective buffer against PTSD symptoms. Mental health services should be embedded into the organizational structure, with accessible and confidential support systems that include therapy, peer support groups, and educational resources. Barriers to accessing care—such as concerns about stigma, time constraints, and lack of coverage—must be addressed by offering paid time for mental health appointments, ensuring confidentiality, and fostering a culture where seeking help is viewed as a sign of strength. Post-trauma interventions must also shift away from ineffective debriefing models toward evidence-based approaches, such as cognitive behavioral therapy, mindfulness training, emotional regulation, and resilience-building programs. Importantly, both formal and informal supports should be recognized as vital components of recovery. Employers should take active steps to strengthen workplace cohesion and encourage social connection outside work, especially for staff at higher risk of isolation, such as junior doctors or newly relocated professionals. Ultimately, prioritizing staff wellbeing through policy reform and supportive practice can help mitigate PTSD risk, reduce burnout, and promote a safer, more sustainable work environment in psychiatric inpatient care.
